# Responses to Microbial Challenges by SLAMF Receptors

**DOI:** 10.3389/fimmu.2016.00004

**Published:** 2016-01-20

**Authors:** Boaz Job van Driel, Gongxian Liao, Pablo Engel, Cox Terhorst

**Affiliations:** ^1^Division of Immunology, Beth Israel Deaconess Medical Center, Harvard Medical School, Boston, MA, USA; ^2^Immunology Unit, Department of Cell Biology, Immunology and Neurosciences, Medical School, University of Barcelona, Barcelona, Spain

**Keywords:** receptors, homophilic, SLAM, SAP, EAT-2, XLP, measles, *Escherichia coli*

## Abstract

The SLAMF family (SLAMF) of cell surface glycoproteins is comprised of nine glycoproteins and while SLAMF1, 3, 5, 6, 7, 8, and 9 are self-ligand receptors, SLAMF2 and SLAMF4 interact with each other. Their interactions induce signal transduction networks *in trans*, thereby shaping immune cell–cell communications. Collectively, these receptors modulate a wide range of functions, such as myeloid cell and lymphocyte development, and T and B cell responses to microbes and parasites. In addition, several SLAMF receptors serve as microbial sensors, which either positively or negatively modulate the function of macrophages, dendritic cells, neutrophils, and NK cells in response to microbial challenges. The SLAMF receptor–microbe interactions contribute both to intracellular microbicidal activity as well as to migration of phagocytes to the site of inflammation. In this review, we describe the current knowledge on how the SLAMF receptors and their specific adapters SLAM-associated protein and EAT-2 regulate innate and adaptive immune responses to microbes.

## Slam Family Receptors and Their Adaptors SAP and EAT-2

### The SLAMF Gene Family

**Figure 1:**
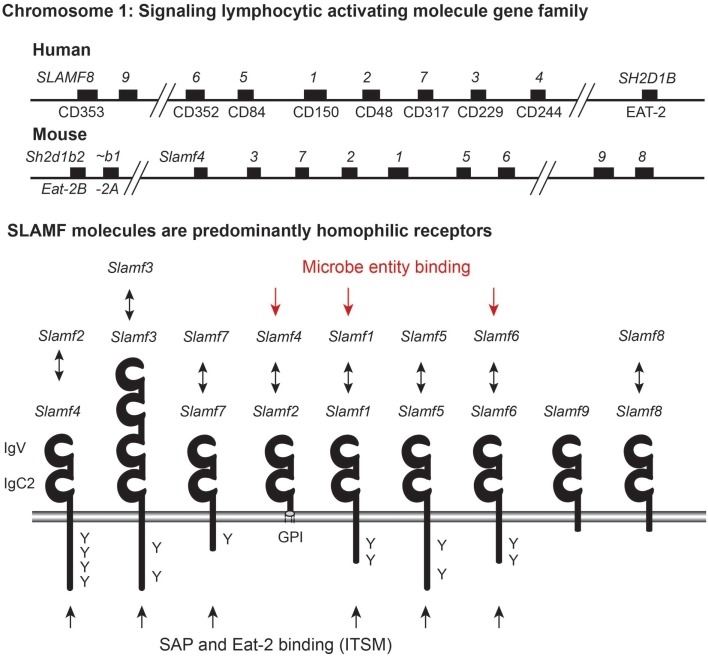
**Signaling lymphocytic activating molecule gene family (SLAMF receptors family) and proteins**. Organizational overview of the SLAM family cluster on chromosome 1 in both human and mice. EAT-2 is also located proximal to this gene cluster and is duplicated in mice, encoding Eat-2a and Eat-2b. The SLAMF receptors are part of the Ig-superfamily and they have an IgV and an IgC2 domain. Seven of the SLAM receptors are homophilic ligands. SLAMF2 and SLAMF4 are co-ligands that bind each other. Three SLAM genes have been shown to possess bacterial binding capacity. Six of the SLAM receptors have docking domains for SAP (and EAT-2) represented by Y (tyrosine in ITSM). SLAMF2 is anchored to the plasma membrane by a GPI-anchor.

**Figure 2:**
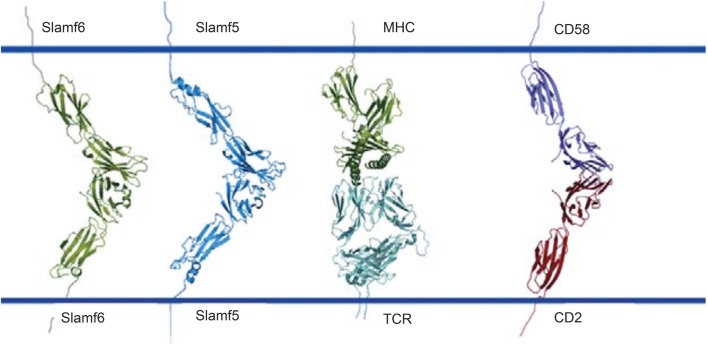
**Ribbon representation of Slamf6 and Slamf5 structures**. Homophilic interactions of SLAMF6 and SLAMF5 as well as heterophilic interactions between two other Ig-superfamily receptors CD58 and CD2. MHC interacting with TCR functions as a reference for the molecular dimensions. Image adopted from Calpe et al. (2).

Seven of the nine members of the signaling lymphocytic activation molecule (SLAM) gene Family (SLAMF1–7), a subfamily of the immunoglobulin superfamily, cluster on the long arm of human and mouse chromosome 1 (1). While SLAMF8 and SLAMF9, as well as the SLAM-associated adaptor EAT-2 (*SH2D1B*) are located in close proximity to the “core” SLAMF locus (shown in Figure 1), the SAP (SH2D1A) gene is on the X-chromosome [reviewed in Ref. (2, 3)]. The nine SLAMF genes encode cell surface receptors, whose expression is mostly confined to hematopoietic cells (Table 1). A wide range of these cells expresses at least one member. The activation state, presence of the adaptor molecules SAP and EAT-2, and the location of immune cells dictate SLAMF receptor expression and function (Figure 2). While SLAMF receptors share intracellular interaction partners and display overlapping features, the individual members of this family have a unique functional signature.

**Table 1:** **Slam receptor expression, associated effector molecules, and functions**.

	Expression	Effectors	SAP-dependent	Eat2-dependent	Other/unknown
SLAMF1, SLAM, CD150	Act T, act B, mono, Mø, DC, plat, HSC	Fyn, Lck, SHIP-1, Src, Shp-1/2, PKCθ, Bcl-10, Beclin-1, PI3K, Nf-κB, Ras-GAP, Akt, JNK1/2, Dok-1/2	T: (+) IL-4, IL-13, proliferation, Th2/Th17 polarization, NKT: development (with Slamf6)	Unknown	T: (+) IFNγ, B: (+) proliferation and activation, (+) apoptosis, Mø: (+) ROS, IL-12, TNFα, NO, (−) IL-6, (+) myeloid cell migration, (+) platelet aggregation, (+) phagocytosis
SLAMF2, CD48	Pan-lymphocyte	Lck, Fyn, RhoA	N/A	N/A	T: (+) IL-2, proliferaton, B: (+) activation, (−) apoptosis Mast: (+) TNFα, eo: (+) activation, mobilization, Mø: (+) TNFα, IL-12, (+) phagocytosis, DC: (+) survival
SLAMF3, Ly-9, CD229	T, B, iCD8, NKT, mono, Mø, HSC	AP-2, Grb-2, ERK, PLZF, NFAT	Unknown	Unknown	T: (−) IFNγ, (+) proliferation, IL-2, IL-4, iCD8^+^ T-cells, iNKT (−) development
SLAMF4, 2B4, CD244	NK, NKT, T, γδ, CD8, DC, eo, mast, mono	LAT, PI3K, Vav-1, SHIP, c-Cbl, ERK, Shp-1/2, PLC-γ, 3BP2, Csk	T: (−) IFNγ, NK/CD8^+^: (+) cytotoxicity, proliferation	NK: (−) Cytotoxicity of Slamf2-neg target cells, (−) IFNγ	eo: (+) adhesion, chemotaxis, peroxidase, (+) IFNγ, IL-4
SLAMF5, CD84	Pan-lymphocyte plat, mast, eo	Dok-1, c-Cbl, ERK, JNK, Fes, Shp-1, Nf-κB	T-B: (+) GC response	NK: (+) Cytotoxicity Mast: (+) Degranulation	lat: (+) spreading
SLAMF6, NTB-A, Ly-108	NK, NKT, T, B, Mø, pDC	PLC-γ, SHIP, Shp-1/2, PI3K, PLZF, Lck, PKCθ, NFAT	T-B: (+) GC response, NK: (+) IFNγ, NKT: development (with Slamf1)	NK: (+) Cytotoxicity	T-B: (−) GC response, Neutro: (+) ROS, (+) IL-6, TNFα
SLAMF7, CRACC, CS1, CD319	T, B, mono, DC, NK	PLC-γ, c-Cbl, SHIP, Akt, Vav-1, Shp-1/2	Unknown/N/A	NK: (+) Cytotoxicity	NK: without Eat2 (−) Cytotoxicity, B: (+) proliferation
SLAMF8, BLAME	iCD8, mono, DC, Mø, Neu, endo, FRC	PKC, p40(phox)	N/A	N/A	(−) myeloid cell migration, (−) ROS, iCD8^+^ T-cells, iNKT (+) development
SLAMF9, SF2001	mono, DC	ND	N/A	N/A	Unknown

*T, T cells; B, B cells; act, activated; Mø, macrophage; DC, dendritic cell; plat, platelet; HSC, hematopoietic stem cell; mono, monocyte; NKT, natural killer T cell; eo, eosinophil; γδ, γδ receptor-expressing T cell; mast, mast cell; endo, endothelial cell; FRC, fibroblastic reticular cell; ROS, reactive oxygen species*.

*Expression data are based on murine expression*.

The consensus structure of SLAMF receptors consists of an extracellular membrane distal IgV domain linked to a proximal IgC2 domain, a transmembrane region, and an intracellular signaling domain that often contains several intracellular tyrosine-based switch motives (ITSM) (Figure 1). Notable exceptions to the consensus structure are SLAMF2, which lacks the intracellular and transmembrane region and instead harbors a glycosyl-phosphatidylinositol membrane anchor; SLAMF3, which has a duplication of the IgV–IgC2 domains; and SLAMF8 and SLAMF9, which only have ~30 intracellular amino acid residues and lack ITSMs.

### Most SLAMF Receptors Are Homophilic

Most SLAMF receptors are self-ligands with signaling motifs, which function in cell–cell communication. Crystal structures of SLAMF1, SLAMF5, and SLAMF6 revealed an angled engagement of the IgV domains *in trans* (4, 5). Exceptions to this homotypic engagement are SLAMF2 and SLAMF4, which are counter-structures (6–8). Ligation of SLAMF receptors leads to inhibitory or activating signaling events through modulation of the cellular responses. Interestingly, SLAMF receptors can also engage microbial structures. For example, SLAMF1 partakes in a xenophilic interaction with the hemagglutinin MH-V of Measles virus, which facilitates viral entry as well as cell fusion (9, 10). As this interaction is thought to benefit the virus, it is *pathogen-centric*. Additional studies also revealed cognate interactions of SLAMF1, SLAMF2, and SLAMF6 with bacterial components (Table 2) (11–13). This class of xenophilic interactions appears to be beneficial for the host and is, therefore, *host-centric*.

**Table 2:** **Slamf receptors and their adaptor SAP modulate susceptibility to microbes**.

	Deficiency: resistant	Deficiency: susceptible	SLAMF ligand	Microbial ligand
SLAMF1	*T. cruzi*	Gram^−^ bacteria, *L. major*	Slamf1	Measles virus, *E. coli* (OmpC/F^+^) *S. typhimurium*
SLAMF2	*S. aureus*	FimH^+^ enterobacterae	Slamf4, CD2	*E. coli (FimH*^+^)
SLAMF3		MCMV	Slamf3	
SLAMF4		LCMV, γHV-68	Slamf2	
SLAMF5			Slamf5	
SLAMF6	*L. mexicana, C. rodentium*	*S. typhimurium*	Slamf6	*E. coli*, *C. rodentium*
SLAMF7			Slamf7	
SLAMF8			Slamf8	
SLAMF9			?	
SAP		Mouse: γHV-68, LCMV, influenza, human: EBV, some other viruses	Slamf1, 3, 4, 5, 6 human: Slamf7	N/A

*SAP (Sh2d1a), SLAM-associated protein; LCMV, lymphocytic choriomeningitis virus; Omp, outer membrane porin; EBV, Epstein–Barr virus; FimH, bacterial lectin; MCMV, murine cytomegalovirus; γHV-68, murine gamma-herpes virus 68*.

*Deficiency: resistant and deficiency: susceptible refer to observations made in Slamf-deficient mice; resistant indicates that knock out animals have milder disease, susceptible indicates that knock out animals have stronger disease manifestations*.

*? Unknown*.

### The SLAMF-Specific Adaptor Proteins SAP and EAT-2

A little under two decades ago, three independent research groups discovered an association between mutations in *SH2D1A*, the gene that encodes the intracellular adaptor protein SLAM-associated protein (SAP) and X-linked lymphoproliferative syndrome (XLP) (14–16). At the same time, we showed that SAP is an intracellular binding partner of SLAMF1, which is required for proper functioning of SAP in response to Epstein–Barr virus (EBV) and other virus. In XLP patients, SAP is mutated or absent resulting in aberrant functioning of SLAMF1 (16).

SLAM-associated protein encodes a small adaptor protein (14 kDa) that consists almost entirely of a Src homology 2 (SH2) domain. SAP can interact with the ITSMs motif of six SLAMF receptors in phospho-tyrosine-dependent and independent modes (Figure 1) (16–19). Mice that are deficient for the gene that encodes SAP (*Sh2d1a*^−/−^) have a range of specific immune malfunctions, which manifest the development and maturation of immune cells and during responses to microbial challenges (20–22). Although SAP expression by T-cells, NK cells, and NKT-cells is well established, B-cells express SAP only under certain specific conditions (23, 24). Some EBV-transformed B-cells, Hodgkin’s lymphomas, and germinal center (GC) B-cells appear to express SAP. The second SLAMF-associated adaptor, EAT-2, exhibits distinct functional features and is not associated with any primary human immune deficiency (25). EAT-2 binds different ITSMs in SLAMF receptors and is involved in the activation of antigen-presenting cells (APCs) and cytotoxicity of NK cells (25, 26). The expression profile of this adaptor also differs from SAP. NK cells express EAT-2 as do a range of APCs, including monocytes (25, 27).

Two SAP signaling modes exist: (1) blockade of the binding of SH2-domain-containing molecules, e.g., the tyrosine-phosphatases SHP-1 and SHP-2 to phosphorylated ITSMs and (2) recruitment of the Src kinase Fyn in its active (“open”) configuration to SAP (3, 16, 28–30). The blocking function of SAP is due to its high affinity for ITSM motifs caused by an unusual three-pronged binding of the SH2 domain (31). In the absence of SAP, SLAMF1 and SLAMF6 bind the tyrosine phosphatases SHP-1 and/or SHP-2, which are negative regulators of T cell functions (16, 17, 32).

**Figure 3:**
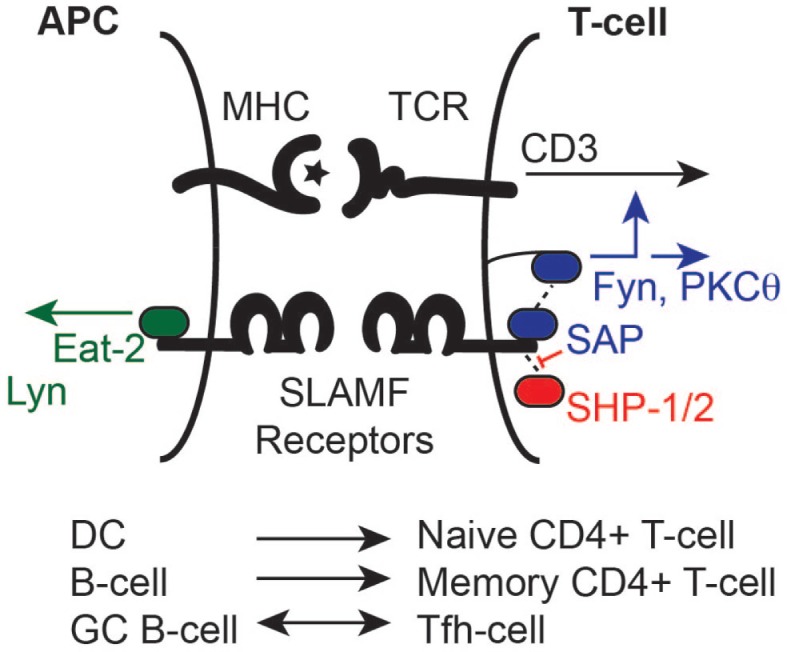
**Slamf receptors modulate the cellular communication between antigen-presenting cells (APCs) and T cells**. Binding of SLAM family members to their ligands induces the phosphorylation of their cytoplasmic tails and the subsequent binding of SLAM-associated protein (SAP) or EAT2 through a tyrosine-containing motif (ITSM). SAP is widely expressed by T cells and EAT2 is expressed by APCs. These two molecules can recruit and activate several Src kinases (including Fyn) that modulate cell activation by signals generated through the T cell receptor (TCR) and costimulatory proteins, such as CD28. Signals mediated by the SLAM receptors can also affect the function of APCs. SLAM receptors recruit various SH2-domain-containing proteins giving rise to different signals that determine distinct and, in some cases, divergent biological outcomes.

A set of functions of SAP in T-cells is dependent on the recruitment of the Src kinase Fyn, which is intricately involved in T-cell receptor (TCR) signaling (Figure 3). SLAMF–SLAMF homophilic ligation leads to the recruitment of SAP to their ITSMs, which interacts with the SH3 domain of Fyn (28, 33). Binding of Fyn to SLAMF1-associated-SAP enhances IL-4 and IL-13 production (29). Structural analyses have shown that Arg78 of SAP is crucial to this interaction (28, 29). Indeed, SAP^R78A^ mice showed a lack of IL-4 production, similar to that of *Sh2d1a*^−/−^ mice (29). Lacking this arginine (28), EAT-2 does not interact with Fyn but associates with a variety of different Src kinases (27). Similar to *Sh2d1a*^−/−^ T-cells, *SLAMF1*^−/−^ CD4^+^ T-cells are also less prone to TCR-mediated IL-4 production (34). It was, therefore, concluded that SLAMF1 contributes to Th2 polarization. Subsequent studies showed that a signaling cascade involving SAP and Fyn as well as GATA-3 transcriptional promotion by NF-κB are responsible for this phenotype (22, 35, 36). This pathway in T-follicular helper cells effectively contributes to GC B-cell maintenance and optimal humoral responses (37).

Overall, these studies have demonstrated that SLAMF receptors and SAP have a complex involvement in mechanisms that fight intracellular infections, via their effect on cytokine production. Together, SAP and EAT-2 dictate the major part of the SLAMF signaling. However, other mediators dictate a distinct set of SLAMF receptor functions.

## Several SLAMF Receptors Interact with Bacteria

### SLAMF1 and SLAMF6 Interactions with Gram^−^ Bacteria

The importance of SLAMF receptors in phagocytes was highlighted by our recent observations that SLAMF1 is involved in cognate interactions with bacterial entities. These interactions result in the defect in the clearance of *Salmonella typhimurium* SseB^−^ after peritoneal infection (11, 12, 38). Thus, direct cognate interactions with microbial components modulate SLAMF functions in phagocytes.

Evidence for direct interactions of SLAMF1 and SLAMF6 with *Escherichia coli* outer membrane porins C (OmpC) and OmpF was shown in a cell-based luciferase reporter assay (11). The specificity of these interactions extends to different Gram^−^ bacteria, but not Gram^+^ bacteria; SLAMF1 interacts with *S. typhimurium* (11); SLAMF6 interacts with *S. typhimurium* and to some degree with *Citrobacter rodentium* (38). Subsequent analyses demonstrated that this interaction depends on the IgV domain of SLAMF1 and SLAMF6. The structure of SLAMF1 has proven difficult to unravel due to the flexible (non-rigid) nature and high degree of glycosylation of SLAMF1. By a combination of techniques, several amino acid residues have been implicated in SLAMF1 homophilic engagement as well as SLAMF1 engagement with Measles virus protein MV-H (10). The FCC beta-sheet and the CC loop of SLAMF1 contain several conserved residues and substitution of Val63, Thr65, Ala67, Lys77, and Glu123 within these regions all resulted in a reduction in the binding of SLAMF1 to SLAMF1 as well as to MV-H. Single mutations of equivalent residues in mouse SLAMF1 resulted in little difference in the binding of OmpC/F containing *E. coli*. In line with this, SLAMF6 engagement with *E. coli* structures does not require amino acid residues in the SLAMF6 IgV domain that are crucial for SLAMF6–SLAMF6 homophilic ligation (38). However, general masking of interaction domains by mAbs directed against epitopes in the IgV domains of SLAMF1 or SLAMF6 blocked their interactions with bacteria (11, 38). Thus, whereas there is overlap in the SLAMF1 residues that are essential for SLAMF1–SLAMF1 ligation with the residues involved in MV-H binding to SLAMF1, it is likely that OmpC/F binding involves a separate set of interacting SLAMF1 residues. This would suggest that the interaction of SLAMF1 with bacteria is of a separate origin, distinct from the SLAMF1–SLAMF1 interaction domain, and hence may represent a SLAMF1 function of separate evolutionary significance. Structural analyses of SLAMF1 or SLAMF6 and *E. coli* outer membrane porins should provide conclusive insights into the mode of these interactions.

### SLAMF1 Enhances Phagocyte Effector Functions

**Figure 4:**
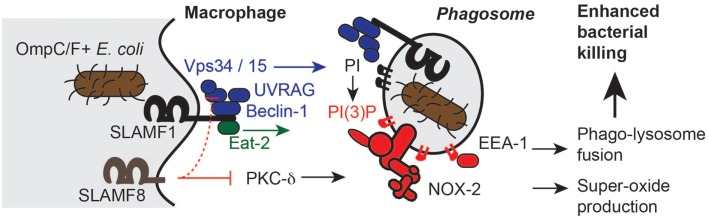
**Slamf1 affects phagosome functions in two ways, after binding to *E. coli***. OmpC/F^+^ *E. coli* can be bound by SLAMF1. Subsequently, SLAMF1 is internalized into the progressing phagosome. The Vps34/15 > UVRAG > Beclin-1 complex is formed. PI is converted to PI3P, which is the docking lipid for subunits of the Nox2 complex as well as the tethering molecule EEA-1. The result of the docking of these proteins is the progression of phagosomes toward bactericidal phagolysosomes that are able to kill the internalized bacteria. The positive modulation of Nox2 complex formation by PKC-delta is inhibited by SLAMF8. There is preliminary evidence for an inhibition by SLAMF8 of Vps34/15 > UVRAG > Beclin-1 complex recruitment to SLAMF1.

The interaction of SLAMF1 with OmpC/F^+^ *E. coli* results in a more effective phagocytosis of these bacteria by macrophages (11). Clusters of SLAMF1 bound to OmpC/F remain proximal to the bacterium during phagocytosis, thus colocalizing to intracellular phagosomes. A signaling complex is recruited to the intracellular domain of SLAMF1 either directly upon bacterial ligation or shortly thereafter during internalization. The transient recruitment of the autophagy scaffold protein Beclin-1 is the initial event that leads to the formation of a functional complex that also contains Vps34, Vps15, and UVRAG (Figure 4) (13). This novel SLAMF1 signaling module is enhanced by, but not prerequisite of the presence of EAT-2 (13). Vps34 supported by its co-enzyme Vps15 is the sole Class III phosphatidylinositol kinase and produces the docking lipid phosphatidylinositol-3′-phosphate (PI_3_P) (39). This SLAMF1-enhanced production of PI_3_P affects two important phagosomal processes. First, formation and activation of the classical phagocytic NADPH oxidase (Nox2) complex is a tightly regulated process that involves assembly of the membrane bound catalytic gp91^phox^ and p22^phox^ with at least four cytosolic subunits p40^phox^, p47^phox^, p67^phox^, Rac1/2 (40). By recruiting the p40^phox^ subunit to the maturing phagosome, PI_3_P initiates the formation of this superoxide-producing complex (39). Second, PI_3_P enables the recruitment of the tethering molecule EEA1, which is critically involved in phagolysosomal fusion. Thus, in the absence of SLAMF1 from phagocytes, the phagocytic process of specific Gram^−^ bacteria is compromised.

### SLAMF2 Interactions with Gram^−^ Bacteria

SLAMF2 is implicated in the recognition of non-opsonized *E. coli* via surface type-1 fimbriae, which contain the lectin FimH (12). Microscopy and genetic analysis suggest that SLAMF2 binds to FimH, which is dependent on the presence of mannose on SLAMF2 (41). Uptake of FimH^−^ *E. coli* is not mediated by SLAMF2 (42).

**Figure 5:**
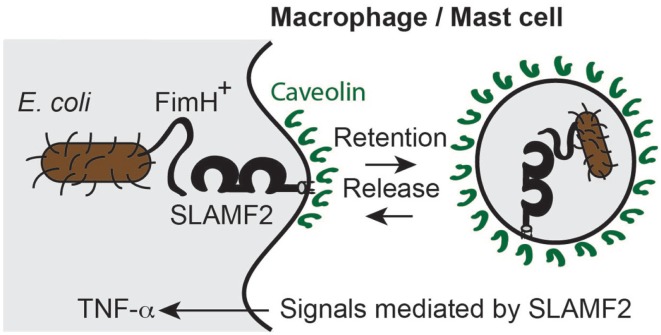
**SLAMF2 mediated the temporary retention of FimH^+^*E. coli* in phagocytes**. SLAMF2 can associate with the bacterial lectin FimH on the flagella of *E. coli*. The bacteria are internalized into caveolin^+^ vesicles to subsequently be released. The presence of SLAMF2 on macrophages and mast cells induced an LPS- or bacteria-mediated enhanced burst of TNF-α production.

SLAMF2 internalizes with FimH upon phagocytosis of FimH^+^ *E. coli* by mast cells and macrophages, which can be inhibited by mAb directed against SLAMF2. The “force catch” interactions between SLAMF2 and FimH are strengthened by the motility that is implicit to fimbriae and, therefore, represents a unique mode of interaction between phagocytes and *E. coli* (43). Studies utilizing mast cells show that the SLAMF2-FimH-mediated phagocytosis, which results in cholesterol-dense *E. coli*^+^ caveolae (44), has a distinct outcome compared to phagocytosis of opsonized *E. coli* (Figure 5). SLAMF2-aided uptake results in the expulsion of the bacterium rather than its intracellular killing (42). Thus, SLAMF2 mediates uptake of FimH^+^ *E. coli* via the formation of caveolin^+^ phagocytes that represent recycling vesicles that release their content to the extracellular milieu within several hours.

### SLAMF Receptors Alter Cytokine Production by Phagocytes

Beside the delayed phagocytosis of *E. coli*, *SLAMF1*^−/−^ macrophages display impaired responses to crude LPS (bacterial homogenate) (11, 13, 34). Stimulation with IFNγ and LPS, but not GpC or PGN, induced an ameliorated production of IL-12, TNF-α, and nitric oxide in *SLAMF1*^−/−^ macrophages (34). Conversely, human DCs that were stimulated with CD40-L expressing cells produced less IL-12 and TNF-α when SLAMF1 costimulation was induced, even in the presence of IFNγ and LPS (45). This discrepancy could suggest that SLAMF1 plays distinct roles on cytokine production in phagocytes, depending on whether SLAMF1 engages in homophilic interactions and/or bacterial interactions (i.e., OmpC/F). Although SLAMF2 has no intracellular signaling domain, SLAMF2 induces signaling events in human brain microvascular endothelial cells that involve an influx of intracellular Ca^2+^ and the phosphorylation of RhoA (46). In mast cells, SLAMF2 engagement results in an increase in their TNF-α production and histamine release (41, 47, 48). Stimulation of *SLAMF2*^−/−^ macrophages with LPS results in reduced induction of TNF-α and IL-12 production (49). No specific interactions of SLAMF5 with bacterial entities have currently been reported, yet SLAMF5 also affects phagocyte functions. Transfection studies in mast cells and macrophages have shown that SLAMF5 signaling enhances phagocyte activation. SLAMF5 engagement induces FcϵRI-mediated mast cell degranulation, which depends on Dok1 phosphorylation (50). Interestingly, LPS stimulation of macrophages results in phosphorylation of SLAMF5 at the second ITSM domain (Y300), which enhances the production of MCP-1 and TNF-α in an NF-κB dependent fashion (51). These observations indicate that SLAMF receptors initiate the signaling through the phosphorylated ITSM motif in phagocytic cells.

EAT-2 may modulate cytokine production. Indeed, recent reports suggest that EAT-2 mediates the production of TNF-α through several SLAMF receptors in human DCs (52). Although specific mechanisms need to be further identified, it is clear that SLAMF receptors modulate inflammatory effector functions of phagocytes in the presence of bacteria or LPS.

## SLAMF8 Inhibits NOX2 Activity in Bacterial Phagosomes

SLAMF8 is a member of the SLAMF receptor family that exhibits unique characteristics, as SLAMF8^−/−^ macrophages appear over-activated. The presence of SLAMF8 in phagocytes inhibits the maturation of phagosomes, irrespective whether the cargoes are Gram^+^ or Gram^−^ bacteria (53). We have recently reported that SLAMF8 negatively regulates the activity of PKC-δ, which phosphorylates the p40^phox^ subunit of the NOX2 complex (53). The presence of SLAMF8, therefore, negatively regulates the production of superoxide. However, the molecular intermediates that facilitate this SLAMF8 function have yet to be determined. Because SLAMF8 does not contain an intracellular domain with known signaling motives, it is unlikely that SLAMF8 recruits adaptor molecules that in turn inhibit PKC. Speculatively, competitive inhibition of SLAMF1 by SLAMF8 represents a possible mechanism. Although interactions *in trans* between SLAMF1 and SLAMF8 did not occur (54), the SLAMF1-Beclin1-Vps34/15-UVRAG complex is more readily formed in the absence of SLAMF8. This preliminary finding alludes to a functional interplay between these two SLAMF receptors.

## SLAMF1 and SLAMF8 Regulate Migration of Myeloid Cells to Sites of Inflammation

### Differential Expression of SLAMF1 and SLAMF8 by Phagocytes

Several SLAMF receptors are highly expressed by phagocytes after activation by inflammatory signals, suggesting a time-sensitive functional significance of SLAMF receptor surface expression in these cells. SLAMF1 expression is induced by stimulation with either LPS or IL-1β and in phagocytes during active colitis (34, 55, 56). Resting blood leukocytes are virtually devoid of SLAMF8 transcripts and protein (57). LPS only marginally induces SLAMF8 expression, rather its expression in phagocytes is mainly dependent on IFNγ signals, which result in a strong upregulation of SLAMF8 (53, 54, 57). Thus, during an ongoing infectious inflammation, phagocytes initially increase SLAMF1 surface expression and subsequently induce SLAMF8 expression.

### SLAMF1 and SLAMF8 Modulate Myeloid Cell Motility

Phagocyte-expressed SLAMF1 positively affects cell migration to sites of ongoing inflammation. Our study that focused on cell motility during inflammation revealed that phagocyte-intrinsic functions of SLAMF1 enhance the capacity to migrate into sites of inflammation (54). Inflammatory phagocytes are required to infiltrate the lamina propria of the colon to establish persisting colitis after transfer of CD45RB^hi^ CD4^+^ T-cell into *Rag1*^−/−^ mice. The impairment of inflammatory phagocytes in *SLAMF1*^−/−^ *Rag1*^−/−^ mice to migrate to the lamina propria, therefore, resulted in ameliorated colitis (55). The poor outcome in SLAMF1-deficient mice of experimental infections with *Leishmania major*, which rely on macrophages for effective clearance, may also be partly explained by impaired migration of macrophage-forming monocytes (34). Opposed to the positive effect that SLAMF1 has on myeloid migration, SLAMF8 has a phagocyte-intrinsic negative effect on cell motility (54). Given the timing of the surface expression of SLAMF1 and SLAMF8 and their opposite effect on phagocyte activation, we hypothesize that these two SLAMF molecules represent a rheostat mechanism that modulates the extent of inflammation at different stages of an infection.

The opposite effects on reactive oxygen production displayed by these two SLAMF receptors were shown to influence cell motility. Specific inhibition of NOX2 activity canceled the *in vitro* migration phenotypes of both *SLAMF1*^−/−^ and *SLAMF8*^−/−^ phagocytes (54). These two phenomena can be linked by the mounting evidence that hydrogen peroxide, which is the more stable intermediate of superoxide, can act as a “second messenger” by oxidizing phosphatases and – as such – modulate cell motility (40, 58, 59).

## SLAMF1, 2, 4, and 6 Regulate Enterocolitis

In line with the observations that SLAMF members modulate the function of phagocytes, three SLAMF receptors (SLAMF1, SLAMF2, and SLAMF6) also affect the pathogenesis of murine models of colitis, which are complex, multifaceted immune events, including activation of the mucosal immune system by microbes. Accumulating evidence by our group and by others shows a role of SLAMF receptors in cognate interactions with bacteria. The infiltration of pro-inflammatory phagocyte into the lamina propria of the colon is also prerequisite of the pathogenesis of colitis and some SLAMF receptors affect the extent of the colitis by influencing this process. Additionally, modulation of cytokine production may also contribute to these colitis phenotypes. No strong intestinal inflammation phenotype has been ascribed to XLP (60), thus SAP-independent functions of SLAMF receptors likely modulate mucosal immune processes.

### SLAMF6 Enhances *C. rodentium* Colitis

*Citrobacter rodentium* are attaching bacteria that harbor a pathogenicity island, which renders them capable of colonizing the colonic epithelia of mice. Colonized *C. rodentium* causes lesions that result in a compromised mucosal barrier. Colitis induced by oral infection with *C. rodentium* is remarkably reduced in mice lacking both the *Rag1* and the *SLAMF6* genes compared to their *Rag-1*-deficient controls, but not in mice that only lack the *SLAMF6* gene (single knock out) as compared to their WT littermates. This shows an involvement of SLAMF6 in innate responses to the mucosal infections with specific enterobacteriae (38). Specific interactions between *E. coli* or *C. rodentium* and SLAMF6 have also been reported. Lacking this interaction in *SLAMF6*^−/−^ mice manifests in impaired functions of phagocytes that first detect the effacing *C. rodentium* bacteria, hence driving the phenotype of reduced pathology (38).

### Phagocyte Functions of SLAMF1 Contribute to Colitis

SLAMF1 in phagocytes also contributes to the development of colitis. By adoptive transfer of CD45RB^hi^ CD4^+^ T-cells into *Rag*^−/−^ or *SLAMF1*^−/−^ *Rag*^−/−^ mice, we found that only SLAMF1 expression by innate cells, and not T-cells, is required for the full induction of experimental colitis (55). Activation of macrophages and DCs via CD40-stimulation alone was not sufficient to overcome the reduced inflammation in *SLAMF1*^−/−^ *Rag*^−/−^ mice, further establishing a phagocyte-intrinsic cause of this phenotype. The hampered migratory capacity of SLAMF1-deficient inflammatory phagocytes was shown to be the primary cause of this phenotype (55). The enhanced phagosomal maturation and ROS production that results from the interaction of SLAMF1 with *E. coli* could represent an additional mechanism if these SLAMF1-mediated functions lead to a higher activation state of the lamina propria phagocytes. The production of pro-inflammatory cytokines that are implicated in colitis development are also impaired by SLAMF1-deficiency (55).

### SLAMF2 Enhances Colitis while SLAMF4 Negatively Regulates Inflammation of the Small Intestine by the Control of Cytotoxic IELs

SLAMF2 is abundantly expressed in all myeloid cells (61). *SLAMF2*^−/−^ T-cells induced colitis in *Rag*^−/−^ mice, but not in *SLAMF2*^−/−^ *Rag*^−/−^ mice, indicating that SLAMF2 expression by both innate cells and transferred T-cells contributes to the development of colitis (49). Indeed, SLAMF2-deficient mice were shown to have severely impaired CD4^+^ T-cell activation and SLAMF2 expression is required on both T-cells and APCs for proper activation (62). Beside T-cell activation, which is a prerequisite for the development of colitis in this model, macrophage-expressed SLAMF2 could contribute to colitis by inducing TNF-α production, as suggested by *in vitro* experiments (41, 49). Whether both SLAMF2 interactions with SLAMF4 and bacteria drive this *in vivo* remains to be determined.

SLAMF4 also affects gut-mucosal immune responses. CD8^+^ T-cell transfer experiments showed that SLAMF4 expression specifically correlated with localization to the intestinal lamina propria, where SLAMF4 modulates homeostasis by negative regulation of the expansion of cytotoxic CD8^+^ IELs (61). SLAMF2 expression in myeloid cells, especially the CX3CR1^+^ and CX3CR1^−^ phagocytes in the lamina propria of the small intestine, facilitates this negative regulation (61). Vice versa, under specific conditions these cytotoxic IELs are capable of controlling the phagocyte population (61).

## SAP and SLAMF Receptors Mediate Protection from EBV and Other Viruses

Whereas SLAMF receptor-mediated immune responses to bacteria are mostly mediated by SLAMF–bacteria interactions, the involvement of SLAMF receptors in antiviral immunity relies mostly on SLAMF–SLAMF homophilic interactions.

### XLP and Epstein–Barr Virus

X-linked lymphoproliferative disease finds its primary cause in dysfunctional SAP (14–16). Often, but not always (63), patients develop fulminant infectious mononucleosis with a fatal outcome upon the first encounter with EBV. Although SAP-deficient patients who survive EBV infections or never encounter EBV will develop aberrant B-cell response such as dysgammaglobulinemia and B-cell lymphomas as well as a lack of innate type lymphocytes such as NKT-cells, the most prominent manifestations of this genetic defect arise in the context of EBV infections. Excellent reviews about EBV-independent immunologic manifestations of the aberrant response in SAP-deficient patients are published elsewhere (3, 64–66). In sum, in the absence of functional SAP, EBV-infected B-cells are not cleared and massive B- and T-lymphocytic expansion is found in most organs. CD4^+^ T-cells, CD8^+^ CTLs, NKT cells, and NK cells are implemented in the defective immune mechanisms that result in uncontrolled or ineffective immune responses to EBV infections in XLP patients. The phenotypic manifestations of non-EBV viral infections in XLP patients are sometimes also more severe than those in SAP-proficient individuals, although the disease manifestations are usually less increased.

### SAP and CD8^+^ T-cell Expansion and Cytotoxic Responses

T-cell receptor signals in naïve T-cells induce a proliferative burst. SAP and SLAMF receptors control both the extent of the CD8^+^ T-cell expansion as well as the cytotoxicity of these cells, thereby influencing the effectiveness of the immune response to viruses as well as potential immunopathology.

In an effort to delineate the complex phenotypes of EBV infections of XLP patients, *Sh2d1a*^−/−^ mice were generated and infected with γHV-68 (67) or LCMV (22, 68). The murine virus γHV-68 is, like EBV and Kaposi’s sarcoma-associated herpes virus, a gamma-herpes virus but has coevolved with rodents and, therefore, does not infect humans. In addition to B-cells, γHV-68 also infects macrophages and DCs, which should be noted when comparing EBV infections of XLP patients with γHV-68 in *Sh2d1a*^−/−^ mice. After infection with γHV-68, *Sh2d1a*^−/−^ mice have an expanded population of CD8^+^ T-cells (69, 70), which produce higher levels of IFNγ as compared to CD8^+^ T-cells from infected WT mice (70). This higher amount of IFNγ controls γHV-68 in macrophages in the peritoneum, but not in the B-cell reservoir (71). In accordance with reports on γHV-68 infected *Sh2d1a*^−/−^ mice, LCMV-Armstrong infections induce a stronger expansion of CD4^+^ and CD8^+^ IFNγ-producing T-cells (22, 68). However, exacerbated immune pathology caused by the over-expansion of CD8^+^ T cells in this infection results in a higher mortality (22, 68).

One of the mechanisms that drive the massive expansion of T-cells is the deregulation of reactivation-induced cell death (RICD). A second TCR activation leads to proapoptotic signals in some expanding T-cells, thereby controlling the extent of the expansion of the collective T-cell pool. XLP patients that suffer fulminant mononucleosis typically lack this T-cell restricting phase of the response to EBV, which is also not observed in virus-infected *Sh2d1a*^−/−^ mice. SAP expression was shown to correlate with the extent of RICD in several cell lines and a lack of cell cycle arrest was found in irradiated lymphocytes from XLP patients (72). The observation that SAP immuno-precipitates with the proapoptotic valosin-containing protein (VCP) alludes to a potential mechanism. A later study showed that SLAMF6 recruitment of SAP and Lck rather than Fyn in these restimulated T-cells results in a proapoptotic signal, which was not observed in T-cells obtained from XLP patients (73).

The expanded population of γHV-68-specific CD8^+^ CTLs in *Sh2d1a*^−/−^ mice does reduce the amount of infected B-cells (69, 70). However, cytotoxicity *per* cell appears not to be affected by SAP (69). In contrast to these murine T-cells, CD8^+^ T-cells from XLP patients are selectively impaired in their cytotoxic response to B-cells (74). These human CTLs showed similar cytokine production and proliferation when they are stimulated *in vitro* with anti-CD3 and anti-CD28 or anti-SLAMF1 mAbs (75, 76). However, incubation with anti-SLAMF4 mAb markedly reduces cytotoxicity of the EBV-specific CD8^+^ CTLs and lowered IFNγ production (76). Because this defect is associated with aberrant lipid rafts, perforin release, and SAP recruitment to the cytolytic synapse, it can be concluded that SLAMF4–SAP pathway plays a critical role in the cytotoxic response of CD8^+^ T-cells to EBV-infected autologous B-cells (75). Indeed, whereas virtually all EBV-specific CD8^+^ T-cells in SAP-proficient individuals are SAP^+^, other viruses induce a mixed pool of SAP^+^ and SAP^−^ virus-specific CTLs (77). The dependence of EBV-specific CD8^+^ T-cells on the SLAMF4–SAP pathway to target infected B-cells together with the narrow B-cells tropism of EBV may represent two of the underlining principles for the strong susceptibility of XLP patients to this virus.

### SAP and CD4^+^ T-Cell Responses and Germinal Centers

Like XLP patients, γHV-68 infected *Sh2d1a*^−/−^ mice had a strong reduction in the amount of GC B-cells (69). These mice also displayed the typical hypo-gammaglobulinemia (67, 69). Whereas SAP-deficient mice develop normal acute IgG responses upon infection with LCMV, they lack a humoral memory response (78). When the (chronic-infectious) LCMV_c113_ strain was used, GCs were grossly absent from *Sh2d1a*^−/−^ mice (68). Lacking adequate help from CD4^+^ T cells, humoral response and cytotoxicity of CD8^+^ T cells are impaired, which renders the immune system not sufficient to clear the virus (68). Protection against secondary influenza infections is best established by CD4^+^ T-cell-mediated humoral responses through the generation of memory B-cells and long-lived plasma cells. Experimental exposure of *Sh2d1a*^−/−^ mice to a second influenza challenge established the observation that these mice have a severely impaired IgG antibody response and, therefore, succumb to this infection (20). Thus, in the late stages of infections with LCMV, γHV-68, and influenza virus, profound defects in humoral immunity become apparent in *Sh2d1a*^−/−^ mice.

SLAM-associated protein is critical for the development of GCs, the anatomical site for B/T-cell cooperation. The observation that T-cell-independent humoral responses are unaffected by SAP deficiency, showed that this phenotype depends on T-cell interactions with B-cells (79). Whereas a B-cell intrinsic SAP component in IgG antibody production was reported in some transfer experiments but not in others, SAP expression by helper T-cells is indispensible for early GC responses (21, 80–82). The contact time of T–B-cell interactions is reduced in SAP-deficient mice, which is the likely underlining mechanism of the impaired GC response (83). Sustained adhesion of T-cells to B-cells is dependent on SLAMF5 (84). An additional study showed that SLAMF6, in the absence of SAP, conveys a negative signal resulting in an insufficient contact time between B-cells and T-cells (32). This negative signal is mediated by SLAMF6 as *SLAMF6*^−/−^ *Sh2d1a*^−/−^ mice (lacking both SLAMF6 and SAP) have normal developing GCs. Recruitment of SHP-1 to SLAMF6 is the signaling event that is responsible for the impaired cognate B/T-cell interaction (32). Although SLAMF1 signaling contributes to GC IL-4 production (37), SLAMF1 and Fyn are not involved in proper GC formation (85). SLAMF3-deficiency does not notably affect GC formation either (86).

### NKT Cell Development Depends on SAP, SLAMF1, and SLAMF6

NKT-cells are implicated in responses to a wide range of microbes and are reactive to lipid antigens. Positive selection of NKT cells is mediated by semi-invariant TCR interactions with lipid antigens in the MHC-I-like CD1d molecule from one double-positive (DP) thymocyte to a neighboring DP thymocyte. Thus, commitment of NKT cells, which takes place in the thymus, is dependent on CD1d stimulation from proximal lymphocytes instead of stromal cells. A secondary signal is required to induce differentiation and expansion. Either SLAMF1 or SLAMF6 homophilic ligation is required for this second signal that induces SAP recruitment to their ITSM (87). SAP-mediated signals are crucial for the development of NKT cells as *Sh2d1a*^−/−^ mice completely lack these cells (88). Upon SAP recruitment to either SLAMF1 or SLAMF6, Fyn binds to the SLAMF–SAP complex to induce signals that facilitate the requirements for differentiation and expansion. In contrast to SLAMF1 and SLAMF6, SLAMF3-deficient mice present elevated numbers of thymic NKT cells, indicating that SLAMF3 plays a unique role as an inhibitory receptor regulating the development of NKT cells (89). An in-depth review of SLAMF receptors in NKT-cells and other innate lymphocyte populations has recently been published (90).

### Role for SAP, SLAMF4, and Other SLAMF Receptors in NK Cells

The capacity of chronic infections with lymphotropic viruses to transform their host cells makes targeted killing of infected cells an important requirement in the immunity to such viruses. SLAMF4 is the major SLAMF receptor to mediate cytotoxicity in both NK cells as well as CD8^+^ CTLs. Initial studies have shown that SLAMF4 interactions with SLAMF2 on target cells induced perforin-mediated killing, which is dependent on SAP (91–95). SLAMF4 phosphorylation is dependent on its sub-location in lipid rafts (96). Within these rafts, association with linker for activation of T-cells (LAT) is prerequisite for SLAMF4 phosphorylation and, hence, SLAMF4-mediated killing of target cells (97). SLAMF4 has four ITSM domains and the membrane proximal ITSM recruits SAP to the cytotoxic immune synapse upon phosphorylation (98). This SLAMF4–SAP complex inhibits the recruitment of inhibitory phosphatases and, hence, is required for a sustained interaction between the NK cell and the target cells (99). However, SLAMF4 can also mediate inhibitory signals in cytotoxic cells (100, 101). The levels of SLAMF4 surface expression on NK cells as well as the abundance of SAP appear to dictate whether signals induce or inhibit targeted killing (95, 102, 103). Naïve human NK cells do not express SAP, but IL-2 or IL-12 stimulation results in the upregulation of SAP expression. Only NK cells that express SAP had the potential to kill target cells by SLAMF4 ligation (104). A recent review describes the intricacies of the dual function of SLAMF4 on cytotoxicity of NK cells in more detail (103).

Whereas SLAMF4 appears to be dominated by SAP, other SLAMF receptors have a stronger dependence of EAT-2. Analysis of EAT-2-mediated signals revealed that EAT-2 induces calcium fluxes and ERK phosphorylation, which results in exocytosis of cytotoxic granules (105). SLAMF6 ligation was shown to induce a cytotoxicity signal by recruiting EAT-2 to its second phosphorylated ITSM, which does not bind to SAP (106). In addition, EAT-2-deficient mice were incapable of SLAMF5- or SLAMF6-mediated targeted killing of SLAMF2^+^ tumors (107). Thus, SLAMF6 signaling through EAT-2 in addition to SAP enhances the cytotoxicity of NK cells. SLAMF7 expression on target cells enhanced NK cell cytotoxicity, which was solely dependent on EAT-2, as *EAT-2*^−/−^ NK cells conveyed a signal that inhibits cytotoxicity through SLAMF7 (26).

## Viral Use of SLAMF Receptors

Thus far, we have discussed how SLAMF receptors perform functions by interactions with bacterial entities and by interaction with SLAMF receptors. SLAMF receptors are also actively targeted by pathogens that seek to use or to alter functions of SLAMF receptors for their benefit. Three such modes of interaction have been postulated to date. First, Morbilliviruses (most prominently Measles virus) utilize SLAMF1 as entry receptors. Second, certain cytomegaloviruses (CMVs) express SLAMF receptors or molecules that closely resemble the structure of SLAMF receptor, potentially representing (negative) competitors of endogenous SLAMF receptors to modulate their functions. Third, several other viruses encode molecules that interfere with cell surface expression of SLAMF receptors and inhibit their functions.

### SLAMF1 on the Surface of Myeloid Cells Binds to the Measles Virus H Protein and Is Involved in Virus Entry

The human pathogenic Measles virus belongs to the lymphotropic Morbillivirus genus. Measles virus and other Morbilliviruses utilize SLAMF1 as one of two entry receptors (9, 108). Crystal structures of SLAMF1 and Measles virus protein MV-H reveal four binding domains that are conserved between marmoset and human but not between mice and human, which determines the tropism of Measles virus (10). Mechanistically, the interaction between SLAMF1 and MV-H reduces the distance between the membranes of the target cell and the virus. The subsequent release of the viral protein MV-F enables fusion of the membranes and, hence, facilitates infection.

**Figure 6:**
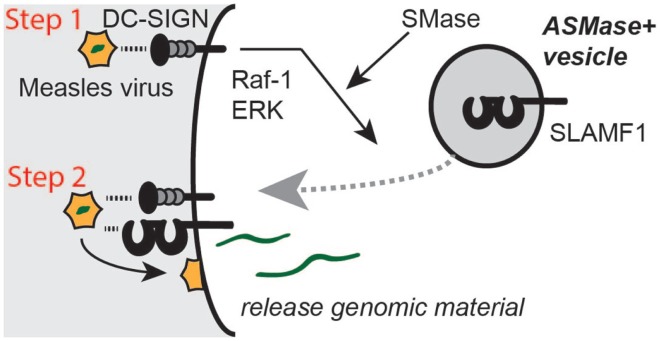
**Measles virus actively recruits its entry receptors Slamf1 to the cell surface**. Binding of the lectin receptor DC-SIGN to a Measles virus particle induces a signaling cascade that involves Raf-1 and ERK and requires the activation of acidic SMase to induce a membrane trafficking event. Slamf1^+^ intracellular vesicles are recruited to the plasma membrane and fuse. This releases Slamf1 to the plasma membrane where Measles viral MV-H protein can bind to it to induce a fusion event between the viral membrane and the plasma membrane, consequently resulting in the delivery of the viral genomic material to the cytosol.

Measles virus has evolved a mechanism to induce SLAMF1 surface expression, thereby gaining access to its entry receptor (109, 110). Acidic Sphingomyelinase (ASMase)-containing vesicles, which are also SLAMF1^+^, play an interesting role in this process (Figure 6). ASMases convert sphingolipids into ceramide, creating a lipid environment that favors endocytosis or internalization of small membrane fractures. Thus, under non-infectious conditions, the recruitment of these vesicles to the surface of cells provides a membrane repair mechanism. Activation of the lectin receptor DC-SIGN by Measles virus induces a signaling cascade that involves Raf-1 and ERK (109). This signal relies on the expression of ASM and results in the relocation of ASM^+^ vesicles to the surface of DCs (109). Thus, by activating DC-SIGN, Measles virus induces surface expression of its entry receptor (110). This observation, thus, provides evidence of a coupling between SLAMF1 localization and membrane dynamics and shows that SLAMF1 resides in intracellular membranes, suggesting that SLAMF1 has distinct intracellular location with putative intracellular functions. These functions may represent events that are similar to the functions that were described for SLAMF1 in *E. coli*^+^ phagosomes.

### Viral Expression of SLAMF Receptor Homologs

SLAMF3 has stronger sequence homology with the human CMV protein UL-7 than with other human SLAMF receptors (111). Only one other CMV, which infects chimpanzees, bears a similar gene, suggesting that this gene was hijacked relatively late during the evolutionary arms race between mammals and β-herpes viruses. While no binding of UL7 to SLAMF3 could be detected, this viral protein has been shown to be secreted from infected cells and to reduce the production of TNFα, IL-8, and IL-6 by DCs (111).

Recently, seven SLAMF gene-homologs encoded by the genomes of two CMVs that infect New World monkeys have been identified. Several of these viral SLAMFs exhibit exceptional preservation of their N-terminal immunoglobulin domains, which results in maintenance of their ligand-binding capacities. The observation that large DNA viruses have captured SLAMF family homologs further underscores the importance of these molecules as critical immune regulators and as convenient scaffolds for viral evolution (112).

### HIV-1 Protein Vpu and CMV m154 Modulate SLAMF Expression

Assessment of SLAMF expression in HIV-1 infected cells showed a negative correlation between SLAMF4 expression by NK cells and viral load, suggesting a positive role for SLAMF4 in the killing of HIV-1 infected cells (113). Indeed, NK cell treatment with specific antibodies for SLAMF4 or SLAMF6 decreased their *in vitro* killing potential of infected T-cells (114). Surface expression of both of these SLAMF receptors is actively down-modulated by HIV-1. CD8^+^ CTLs of patients required both SLAMF2-to-SLAMF4 signaling and TCR stimulation for the downmodulation of SLAMF4 surface expression (115). HIV-1 infection also down-modulates the expression of SLAMF2 and SLAMF6 in infected CD4^+^ T-cells, suggesting active modulation of cytotoxicity by the virus. The HIV-1 protein Vpu associates with SLAMF6 by interacting at the transmembrane regions. This interaction interferes with the glycosylation of SLAMF6 and results in retention in the Golgi-complex (116, 117). SLAMF6 downmodulation leads to insufficient degranulation, and hence impaired targeted killing of HIV-1 infected cells (116).

Murine CMV encodes a different viral protein that interferes with NK cell cytotoxicity. During CMV infection, m154 expression leads to proteolytic degradation of SLAMF2 that reduces the capacity of NK cells to kill infected cells (118).

### Detrimental Effects of SLAMF4 During Chronic Hepatitis Infection

Lysis of non-MHC HCV-infected cells by activated CD8^+^ T-cells is mediated by SLAMF4 (119). However, during chronic HCV infections, SLAMF4 predominates as an inhibitor of cytotoxic functions in CD8^+^ T-cells (95). In line with this notion, recombinant IFN-α therapy of HCV-infected patients induces NK cell-mediated enhanced immunity but reduces SLAMF4 expression of these cells (120). SLAMF4 expression by CD8^+^ T-cells also correlated with poor clinical outcomes in HBV-infected patients (121). Blockade of SLAMF4 signaling effectively enhanced IFNγ production and virus-specific CD8^+^ T-cell proliferation in approximately one-third of HCV^+^ patients (122). Overall, SLAMF4 expression correlates with the T-cell exhaustion that is typically observed during HCV infections. However, functionally exhausted T-cells are not universally revived by blockade of SLAMF4 alone, but other CTL inhibitory receptors are involved (122). Thus, these β-herpes virus infections cause the expression and function of specific SLAMF receptors to be detrimental to the immune outcome.

## Concluding Remarks

SLAMF receptors and their adaptors are intricately involved in the responses to microbial challenges. Modulation of immune responses as a result of SLAMF receptor homophilic interactions represents an important category of functions for these receptors. We can also observe an emerging theme that places SLAMF receptors in a possibly underappreciated category of functions; they can engage microbial ligands. SLAMF receptors are direct microbial sensors and are part of functional anti-microbial mechanisms. Thus, SLAMF receptors fulfill a unique role within the immune system, as they are both microbial sensors and cell–cell communicators of immunologic conditions. Additionally, we can distinguish a category of microbe-encoded genes that directly interfere with SLAMF functions. Interestingly, some of these genes have strong homology with endogenous SLAMF receptors.

## Author Contributions

BvD, CT: initial writing and collection of literature. GL: writing and editing. PE: expertise on virus – SLAMF interactions, editing.

## Conflict of Interest Statement

The authors declare that the research was conducted in the absence of any commercial or financial relationships that could be construed as a potential conflict of interest.
